# Fe_3_O_4_–Silicone Mixture as Flexible Actuator

**DOI:** 10.3390/ma11050753

**Published:** 2018-05-08

**Authors:** Kahye Song, Youngsu Cha

**Affiliations:** Center for Intelligent & Interactive Robotics, Korea Institute of Science and Technology, Hwarangro 14gil 5, Seongbuk-gu, Seoul 02792, Korea; kahye@kist.re.kr

**Keywords:** soft active materials, flexible actuator, electrostatic force

## Abstract

In this study, we introduce Fe_3_O_4_-silicone flexible composite actuators fabricated by combining silicone and iron oxide particles. The actuators exploit the flexibility of silicone and the electric conductivity of iron oxide particles. These actuators are activated by electrostatic force using the properties of the metal particles. Herein, we investigate the characteristic changes in actuation performance by increasing the concentration of iron oxide from 1% to 20%. The developed flexible actuators exhibit a resonant frequency near 3 Hz and their actuation amplitudes increase with increasing input voltage. We found that the actuator can move well at metal particle concentrations >2.5%. We also studied the changes in actuation behavior, depending on the portion of the Fe_3_O_4_-silicone in the length. Overall, we experimentally analyzed the characteristics of the newly proposed metal particle-silicone composite actuators.

## 1. Introduction

Flexible actuators are usually made of flexible materials such as polymers [1]. Thus, such actuators have various functional advantages, including their light weight and flexibility. Further, flexible actuators have a high strain density and are easy to fabricate to desired shape [2,3].

Flexible actuators can deform and move in response to external stimuli [4]. During operation, flexible actuators exhibit a large macroscopic actuation with little stimulation [1,5]. They allow straight axial and curved movements in one or more planes. Further, they compromise to achieve complex motions and serve in diverse movement-related platforms [1,6]. In particular, flexible actuators with relatively simple mechanisms perform complex motions that can be handled by complicated control systems and large-scale components of hard machines [5,6]. Thus, flexible actuators have been utilized in various fields, including medical and wearable applications. Specifically, flexible actuators are excellent for rehabilitation and restoring a patient’s movements [3,7,8]. Furthermore, flexible actuators may be necessary for soft robot parts, such as soft robotic hands, in multi-segment continuum robots, and miniaturized drilling devices [9,10]. To satisfy these needs, various materials have been adapted to develop flexible actuators.

In general, flexible actuators can be divided into two main categories: actuators driven by an electric field, called electroactive polymer (EAP) actuator, and those driven by other stimuli, including optical, thermal, and chemical stimuli [4]. In addition, EAP actuators can be generally divided into two main classes: dielectric and ionic [11]. In dielectric elastomer actuators, a field-induced activation reaction is triggered by electrostatic attraction between the two charged conductive layers applied to the surfaces of the polymer membrane [12]. A voltage potential difference is applied between the two compliant electrodes, causing reduction of thickness and increase of area of the polymer film. Ionic EAP actuators work by migration of mobile ions within the polymer [4,13,14]. They exhibit discontinuous changes due to small changes in the external variables such as electric and magnetic fields, temperature, solvent quality, and pH. Examples of ionic EAPs include polymer electrolyte gels, ionic polymer–metal composites (IPMCs), conductive polymers, and bucky gel actuators [4].

Although all flexible actuators have different operating methods and material properties, all of them exhibit a high degree of flexibility. Furthermore, research on novel materials and fabrication techniques is continuously being undertaken [5]. Recent advancements in materials have enabled the development of high reliability and performance actuators [15]. For example, a study was conducted to improve the thermal stability and mechanical performance of actuator materials [16]. A recent research also suggests new process methods for reproducible and fast actuators with long life spans [17]. With such researches, new types of actuators have been reported. For instance, multilayer dielectric elastomer actuators based on silicone materials and elastomer electrodes have been studied with many advantages in terms of thickness and manufacturing [18]. In addition, millimeter-scale cylindrical actuators have been developed with silicone polymer, liquid metal (LM) alloy (eutectic gallium indium, EGaIn), and magnetic (NdFeB) powder [19]. Also, actuators with carbon nanotube network in silicone elastomer showed excellent performance under low voltage range [20,21]. Likewise, in this study, we introduce new flexible actuators based on composite structures comprising highly elastomeric siloxane (Ecoflex) and iron oxide.

Silicone rubber has an average modulus of elasticity of several hundred KPa, a Poisson’s ratio of 0.49–0.50, and a shear modulus of several tens kPa [22,23]. In case of Ecoflex, a commercial silicone rubber, its elastic modulus is 125 kPa [22]. In addition, because silicone is harmless to the human body, soft silicone or porous silicone is used as a surgical material and in daily life [24]. However, silicone does not actuate by electrical stimulation. Herein, we added iron oxide particles to silicone rubber to control it as an actuator with electric input. Iron oxides are common and easily synthesized compounds [25]. Among the various types of iron oxide available, Fe_3_O_4_ is a strong electrical conductor with a higher conductivity than Fe_2_O_3_ at temperatures above 120 K (10^2^ to 10^3^ Ω^−1^·cm^−1^) [26]. In composites consisting of a polymer, heterophasic polypropylene copolymer, polypropylene block copolymer, and polyamide 6, which are considered as insulators, and Fe_3_O_4_ particles, electrical conductivity can be varied by more than seven orders depending on the mixing ratio [27].

We experimentally demonstrate how the beam type of the Fe_3_O_4_-silicone composite actuator reacts to high-voltage input. Further, frequency resonance with respect to the largest motion is experimentally studied. In addition, we try to understand the changes in actuation performance with an increase in the concentration of metal particles and attempt to understand the principle behind actuation.

## 2. Materials and Methods

### 2.1. Beam Shape Mold Fabrication Using 3D Printer

A mold for beam-shaped specimens (50 mm length × 5 mm width × 1 mm thickness) was designed using Solidworks software (Dassault Systems Solidworks Corp., Waltham, MA, USA). Specifically, the mold was made using a 3D printer (ProJet HD3500, 3D Systems Inc., Rock Hill, SC, USA). The designed mold was made of part (VisiJet M3 Crystal, 3D Systems Inc., USA) and supporter (VisiJet S300, 3D Systems Inc., USA) materials. After printing, the mold was heated in a convection oven (DCF-31-N, Dae Heung Science, Incheon, Korea) for melting the supporter. Lastly, the supporter was completely removed from the mold in an oil bath in an ultrasonic cleaner (Sae Han Ultrasonic Co., Seoul, Korea). After washing and drying, a release agent (Ease release 200, Smooth-On, Inc., Macungie, PA, USA) was sprayed on the mold surface to prevent the silicone from sticking to the surface of the mold.

### 2.2. Iron Oxide-Silicone Composite Beam Fabrication

The basic fabrication method of Fe_3_O_4_-silicone composite actuators is similar to the general method used for the fabrication of platinum-catalyzed silicones rubbers. Firstly, Ecoflex 0030 part A (Smooth-On, Inc., USA), Ecoflex 0030 part B (Smooth-On, Inc., USA), and platinum silicone cure accelerator (Plat-cat, Smooth-On, Inc., USA) were mixed at a ratio of 1:1:0.04 for 3 min. Extra pure tri-iron tetra-oxide powder (Daejung Chemical & Metal Co., Ltd., Siheung, Korea) was added to this mixture (in this study, as much as 1–20% of the total weight) and mixed. The well-mixed and homogeneous mixture was then poured into the fabricated mold and cured at room temperature for 2 h. The actuator specimens thus fabricated were in the shape of a beam of dimensions of 50 mm length × 5 mm width × 1 mm thickness; the specimens were carefully separated from the mold using tweezers after they were fully cured.

### 2.3. SEM Images and Microanalysis

To observe the details of the microstructure of the actuators in terms of the iron oxide concentration and distribution, field-emission scanning electron microscopy (FE-SEM, Inspect F50, FEI, Hillsboro, OR, USA) was carried out. The samples were immersed in liquid nitrogen to preserve the internal structure without crushing the cross section. The ends of the instantly frozen samples were held with tweezers and the samples were broken in two. The samples were fixed with a carbon tape to the SEM sample holder and observed in back-scattered electrons (BSE) mode to analyze the distribution of iron oxide. Further, microanalysis was carried out to analyze the type of metal represented by the bright points seen in the SEM images.

### 2.4. Circuit Configuration and Setup

A high-voltage converter (AG 50P-5, XP Power, Singapore) was supplied from a power supply (MK3003P, MK Power, Seoul, Korea) and controlled as a sinewave function using a waveform generator (33500Bseries, Keysight Technologies, Santa Rosa, CA, USA), from DC 3 kV ± AC 1 kV to DC 3 kV ± AC 2 kV. A thick film resistor (50 M ohms, Ohmite, Warrenville, IL, USA) was also connected to the output of the converters for electric charge release.

An aluminum plate with the same surface area (50 mm × 5 mm) as the composite actuator was prepared. A polyethylene terephthalate (PET) film (Toray, Seoul, Korea), 100 μm thick, was attached to the overall surface of the aluminum plate to prevent electric shorting during actuator operation. The aluminum plate and the actuator were positioned 1 cm away from each other in a parallel configuration. Copper tape (1181, 3M, Saint Paul, MN, USA) with a soldering wire was utilized to form the electrodes. They were connected to the output of the converter ((+port): Fe_3_O_4_-silicone composite actuator, (−port): aluminum plate).

### 2.5. Motion Tracing of the Silicone Composite Actuator

Positional changes in the Fe_3_O_4_-silicone composite actuator were detected using a point laser sensor (IL-100 Intelligent Laser sensor, Keyence Corp., Osaka, Japan). The laser sensor data was amplified using an IL-1000 amplifier unit (Keyence Corp., Japan). The detecting point of the sensor was located 3 mm above the tip of the actuator. The output of the laser sensor was measured at a sampling frequency of 1000 Hz using a data acquisition board (USB-6343, National Instruments, Austin, TX, USA). In addition, the motion of the Fe_3_O_4_-silicone composite actuator was monitored using a 4 K camera (DSC RX10M3, Sony, Tokyo, Japan with a Vario-Sonnar T* lens, Zeiss, Oberkochen, Germany) at a framerate of 240 in high framerate (HFR) mode. The generated series of photographs was analyzed using the Image J software (National Institutes of Health, Bethesda, MD, USA).

## 3. Results

### 3.1. Material Properties of the Fe_3_O_4_-Silicone Composite Actuators

**Figure 1:**
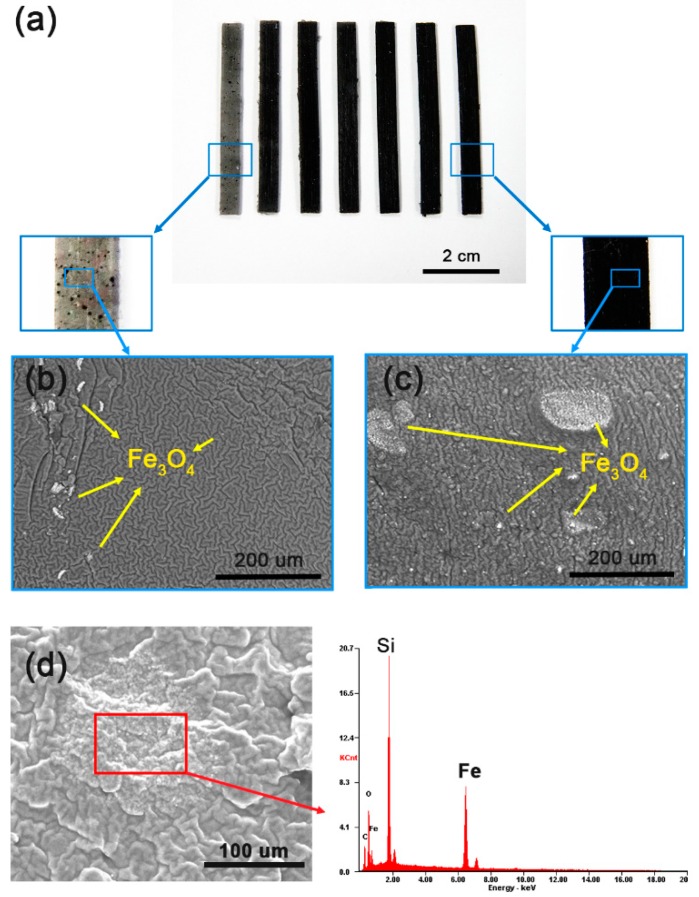
Characteristics of Fe_3_O_4_-silicone composite actuator depending on iron oxide concentration. (**a**) Fe_3_O_4_-silicone composite actuator beams with 1% to 20% iron oxide concentration (from left to right: 1%, 1.5%, 2%, 2.5%, 5%, 10%, and 20%). SEM images of Fe_3_O_4_-silicone composite actuators: (**b**) 1% and (**c**) 20%. The dark gray wrinkled structures represent silicone, while the bright dots represent iron oxide particles. (**d**) Microanalysis results show that the bright dots correspond to iron oxide.

Silicone rubber is a translucent material [28]. When it is mixed with iron oxide particles, as the concentration of the iron oxide particles increases, the color of the material gradually becomes darker (Figure 1a). Numerous nano-sized iron oxide particles are scattered in silicone rubber. At low iron oxide concentrations, the material appears spotted. At concentrations greater 2.5%, the iron oxide particles are well distributed and the actuator looks like a black beam. We fabricated seven samples with varying iron oxide concentration from 1% to 20%. In preliminary testing, it was found that when the concentration of iron oxide is greater than 25%, they can no longer be mixed with silicone.

We observed an enlarged cross-section in the BSE mode of SEM analysis (Figure 1b,c). Shiny lumps, a few micrometers to several hundred micrometers in size, are positioned in the silicone matrix. In the actuator with a low iron oxide concentration of 1%, iron oxide lumps are observed only on some sides, but in an actuator with a high iron oxide concentration of 20%, lumps are distributed evenly on most parts. The higher the concentration of iron oxide particles, the greater the distribution of iron oxide masses. Microanalysis confirmed that the shiny bright spots between the silicone chains corresponded to iron (Figure 1d).

### 3.2. Motion of Fe_3_O_4_-Silicone Composite Actuators

#### 3.2.1. Actuation by High-Voltage Input

**Figure 2:**
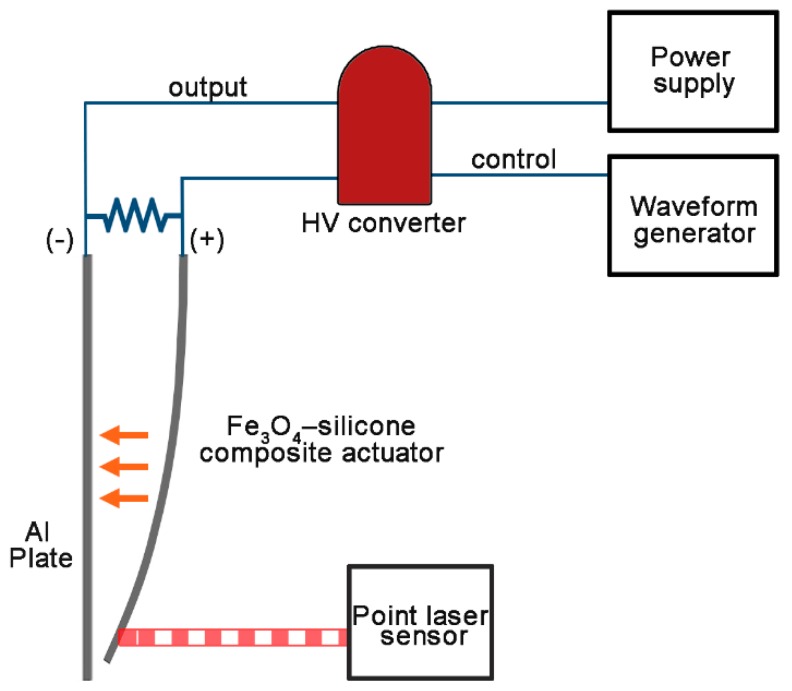
Schematic of the circuit configuration and experimental set up. A high voltage is supplied to Fe_3_O_4_-silicone composite actuators and controlled using a waveform generator. The + and − ports of the high voltage output are connected to the actuator and aluminum plate, respectively. The distances moved by the actuator are measured using a point laser sensor.

**Figure 3:**
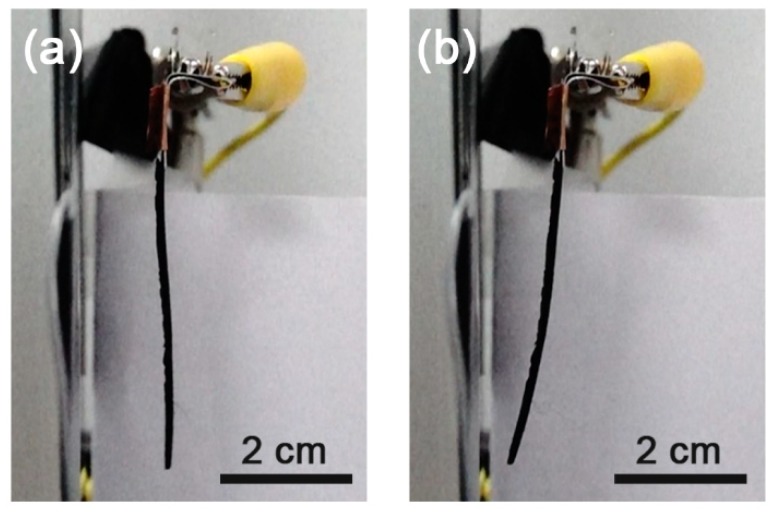
Motion of a Fe_3_O_4_-silicone composite actuator with an iron oxide concentration of 10% (240 fps). (**a**) Off-state and (**b**) on-state.

The fabricated actuator was placed in parallel to an aluminum plate of the same size (Figure 2) and they were connected to each other through different ports of the high voltage output ((+): Fe_3_O_4_-silicone actuator, (−): aluminum plate). When a voltage potential was supplied to the actuator and aluminum plate, the actuator started moving towards the aluminum plate (Figure 3a,b).

#### 3.2.2. Frequency Response and Effect of Voltage input

**Figure 4:**
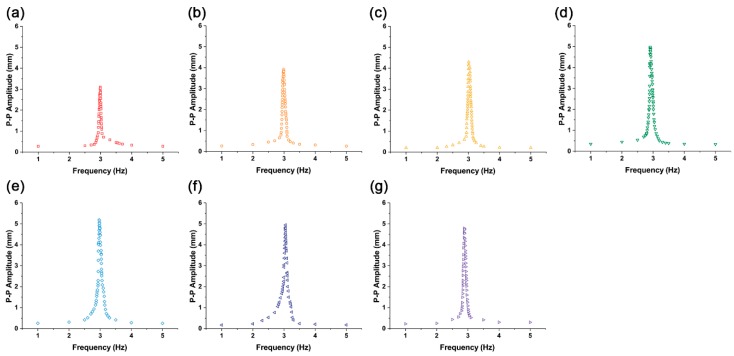
Frequency responses of Fe_3_O_4_-silicone composite actuators with different iron oxide concentrations. (**a**) 1%, (**b**) 1.5%, (**c**) 2%, (**d**) 2.5%, (**e**) 5%, (**f**) 10%, and (**g**) 20%.

Actuators with iron oxide concentration in the range of 1% to 20% were tested. Figure 4 illustrates how the peak-to-peak amplitudes change with frequency when the input voltage is varied. All actuators show a peak corresponding to their resonance frequency.

**Figure 5:**
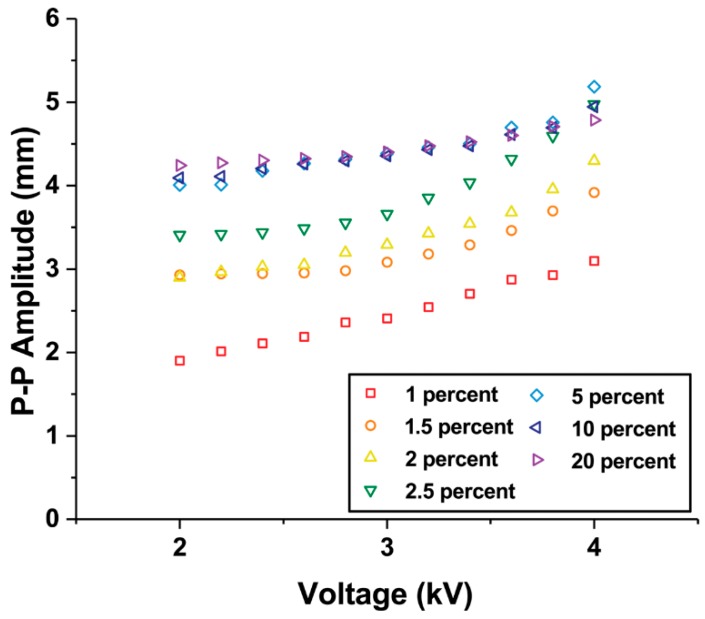
Peak-to-peak amplitude changes observed upon varying the input peak-to-peak voltage values from 2 kV to 4 kV in Fe_3_O_4_-silicone composite actuators with different iron oxide concentrations.

Additionally, we varied the magnitude of the applied voltage input at the resonant frequencies of the fabricated composite actuators. As the voltage increases, the peak-to-peak amplitudes of all the actuators tended to increase gradually (Figure 5). The slope of the actuator with an iron oxide concentration of 2.5% is the steepest.

**Figure 6:**
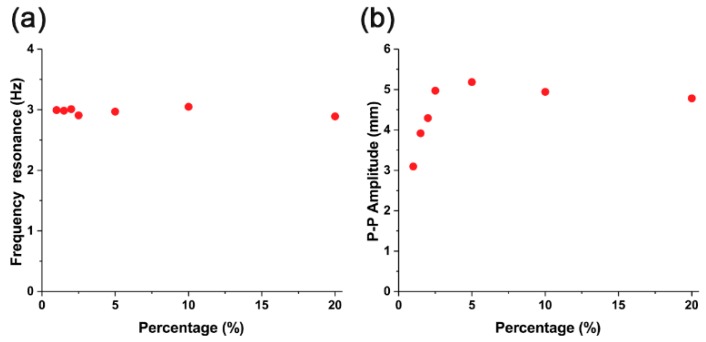
(**a**) Frequency resonance and (**b**) peak-to-peak amplitude of Fe_3_O_4_-silicone composite actuators.

In terms of the frequency response, all actuators show a constant frequency resonance of 3 Hz ± 0.1 Hz, regardless of the iron oxide concentration (Figure 6a). In the concentration range of 1% to 2.5%, the peak-to-peak amplitudes increased (Figure 6b). However, when the iron oxide concentration was over 2.5%, the peak-to-peak amplitude saturated. At an iron oxide concentration of 5%, the highest peak-to-peak value of 5.19 mm was observed. After this point, the peak-to-peak value decreased slightly.

The Young’s modulus of the actuators can be calculated as follows [29],

(1) $$E=38.3\times \rho \times \frac{f_{r}{}^{2}\times L_{m}{}^{4}}{T^{2}}$$

where *ρ* is the mass density (kg/m^3^); *f_r_* is the resonant frequency (Hz); *L_m_* is the movable length of the beam (m); and *T* is the thickness of beam (m). The movable length is 4.5 × 10^−2^ m because the clamped distance of the actuator is 5 × 10^−3^ m. The elastic modulus can be calculated by substituting the measured parameters (*ρ* = 1240 kg/m^3^) and the experimental results in Figure 6. The calculated elastic modulus of the actuators is 1.75 MPa ± 0.11 MPa. This is presumably because the iron oxide concentration ranges from 1% to 20% (by weight) and corresponds to 1.11% in volume. This does not have a huge impact on Young’s modulus.

## 4. Discussion

In this study, we fabricated composite actuators by combining silicone and iron oxide particles and investigated their characteristics. The actuators were very active at their resonant frequencies.

**Figure 7:**
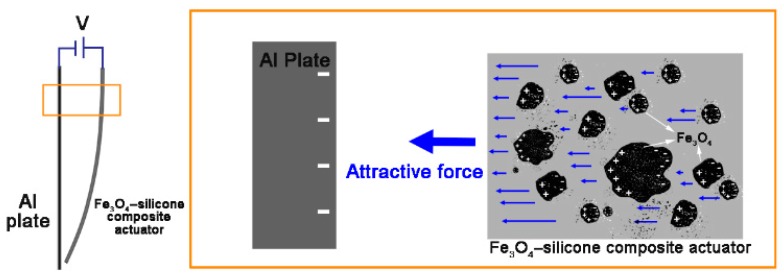
Surface of the aluminum plate (where the negative potential is applied) is conducted to the negative electrode. On the other hand, when a positive potential is applied to the actuator, the iron oxide particles near the top are conducted to the positive electrode. Thus, electrostatic attraction is generated, and the actuator moves.

Herein, we discuss the operation principle of the developed Fe_3_O_4_-silicone composite actuators. The iron oxide particles are not regularly arranged in the actuator, but are actually scattered as lumps (Figure 1b,c). Particularly in the case of the actuator with 1% iron oxide, the lumps are barely noticeable (Figure 1b). Further, when we tried to measure its resistance using a multimeter, it was beyond the measurement range. Therefore, it is suggested that the iron oxide particles inside the actuator are not connected to each other, and it is difficult for them to be electrically connected to the tip of the actuator. Thus, the motion can be explained by the concept of an internal field between the electrode and the adjacent particles [30]. The concept of internal fields links molecular and macroscopic characteristics. The difficulty in determining the electric field acting on a single dipole in the dielectric can be attributed to its dependence on the polarization of neighboring molecules. Therefore, the basic concept is to consider spherical regions containing dipoles in the dielectric. In a ferroelectric polymer, dipoles can be randomly oriented, but the application of a high electric field causes the dipoles to align and thus exhibit a ferroelectric behavior [4,31]. Spheroidal iron oxide particles contain dipoles and the dielectric polarization direction is parallel to the external electric field. Polarization occurs in the vicinity of the top region, which is close to the positive potential electrode (Figure 7). At the tip of the composite beam, it is difficult to induce polarization due to discontinuous conductivity along the length axis. Therefore, the polarized conductive masses in the top region tend to adhere to the aluminum plate, which has opposite polarity. In terms of beam actuation, the electrostatic force in the top region is the reason for motion. The bottom region of the actuator follows the motion of the top region.

**Figure 8:**
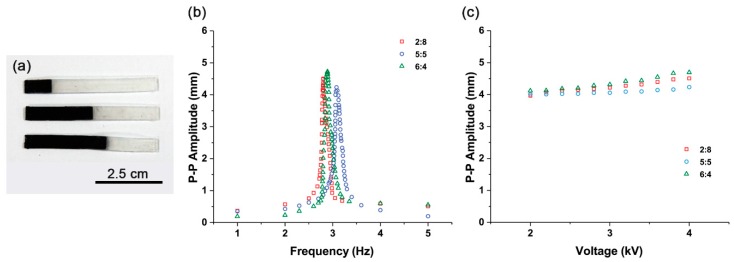
(**a**) Composite actuators with pure silicone-Fe_3_O_4_ and silicone mixture. From top to bottom: 2:8, 5:5, and 6:4. (**b**) Frequency responses of pure silicone-Fe_3_O_4_ and silicone mixture composite actuators with different Fe_3_O_4_ and silicone mixture portions. (**c**) Peak-to-peak amplitude changes observed at different voltage input peak-to-peak values from 2 to 4 kV in pure silicone-Fe_3_O_4_ and silicone mixture composite actuators with different Fe_3_O_4_ and silicone mixture portions.

In other words, even if there are no iron oxide particles at the bottom of the actuator, it functions as an actuator. Additional experiments were conducted to demonstrate this phenomenon. Actuators with partially compound Fe_3_O_4_-silicone with 10% iron oxide concentration were fabricated (Figure 8a). Parts (80%, 50%, and 40%) of the total length of these samples were occupied by pure silicone. Experimental results show that the actuators are actively driven by the Fe_3_O_4_ and silicone mixture mixed in proportions of not only 50% but also 20% (Figure 8b). Further, the peak-to-peak amplitude changes with increasing voltage input and exhibits a similar trend (Figure 8c). We observe that there is no significant difference between the samples in which iron oxide is partially occupied and the sample in which Fe_3_O_4_ and silicone mixture is uniformly distributed throughout (Figure 4f and Figure 5).

## 5. Conclusions

In this study, we fabricated flexible actuators using combinations of silicone rubber and iron oxide particles. Specifically, the metal oxide particles exhibit a ferroelectric behavior and generate movements in the composite in the vicinity of the electrode. We analyzed the effect of the concentration of iron oxide particles by a series of experiments and found that the actuation performance is improved up to a concentration of 2.5%; there is no significant effect of iron oxide particles beyond this concentration. In addition, we tested the actuators with partial compositions of metal oxide particles and silicone. We observed that the actuator moves well even if only 20% of the total length is occupied with Fe_3_O_4_ and silicone mixture.

The developed actuators and their fabrication methods can be employed in various ways. Due to the inherent flexible and moldable nature of silicone, it is easy to manufacture it in various shapes. Moreover, it is possible to generate actuation with a small amount of metal particles. Flexibility and the strength of being able to make it in any form can be applied in various fields. It can be used as valves in microchannels and various medical devices including robotic grippers. Additionally, it can be utilized in industrial fields including robot manipulators and weighting machines. In addition, as iron oxide is mixed, it can be used for developing actuators stimulated by both electric and magnetic fields [32].
